# Subdural empyema—a rare complication of chronic otitis media: a case report

**DOI:** 10.1186/s13256-024-04671-4

**Published:** 2024-08-03

**Authors:** Emnet Tekeste Fekadu, Nahom Daniel, Samuel Tekle Mengistu, Genet Tekeste Fekadu

**Affiliations:** 1 Barentu Military Hospital, Barentu, Eritrea; 2 Mendefera Zonal Referral Hospital, Mendefera, Eritrea; 3 Nakfa Hospital, Ministry of Health Northern Red Sea Branch, Nakfa, Eritrea; 4 Orotta National Referral Hospital, Asmara, Eritrea

**Keywords:** Subdural empyema, Chronic otitis media, Case report, Eritrea

## Abstract

**Background:**

Subdural empyema is an extremely rare and fatal intracranial complication of chronic otitis media. Due to its rarity and vague symptoms, it is often diagnosed late if not completely missed; specially in developing countries where the diagnostic modalities are hardly available or accessible. To the best knowledge of the authors, this is a preliminary reported case of subdural empyema as a complication of chronic otitis media in Eritrea. It aims to provide vital information on the clinical presentation, preferred diagnostic modalities, and the proper management of such cases.

**Case report:**

An 8 years old female patient from the Rashaida ethnic group presented with fever, right ear purulent discharge, right post-auricular swelling, and altered mental status. Prior to her admission, she had history of recurrent purulent discharge from her right ear for almost 2 years, and had been diagnosed with chronic otitis media. Upon admission her GCS was 13/15 which later on deteriorated to be 3/15 on day 3. MRI was done and showed a right fronto-tempo-parietal subdural empyema with mass effect, shifting the midline to the left. She was immediately started on empirical broad-spectrum antibiotics. After the diagnosis was made, craniotomy was done, and 30 ml of pus was removed from the subdural space. Culture and sensitivity of the pus obtained intraoperatively was done but produced no yield. Hence, she was continued on the empirically started antibiotics. The patient’s condition was well improved by post-operative day 4.

**Conclusion:**

It is important to have a high index of suspicion of intracranial complications in patients with history of chronic otitis media or other otologic complaints, who present with neurologic manifestations. Subdural empyema still being uncommon even among the intracranial complications of COM, it is often missed. Hence, timely diagnosis with MRI, immediate surgical evacuation of the empyema along with the prolonged administration of broad-spectrum antibiotics is highly recommended.

**Supplementary Information:**

The online version contains supplementary material available at 10.1186/s13256-024-04671-4.

## Introduction

Otitis media is a disease of the middle ear marked by the inflammation of the mucosal lining. When it lasts more than 2 weeks, it develops into chronic otitis media (COM). Chronic otitis media is marked by the presence of a perforated tympanic membrane and recurrent purulent ear discharge [1–3]. Otitis media is one of the most common infections of childhood: that by the age of 3 years more than 80% of children will have had at least 1 episode of otitis media [4]. COM can lead to both extracranial, and intracranial complications. Sixty to seventy percent of these complications occur in children and young adults: that is, during the first two decades of life [5–7]. Intracranial complication is an infrequent complication with reported rates ranging from 0.3% in developed countries to 3% in developing countries [5, 6]. Its pathogenesis, that is its spread from the ear canal, is through direct invasion of the bone, thrombophlebitis, haematogenous spread, or dissemination through congenital or traumatic bone defects [6, 8, 9].

The most common intracranial complications are brain abscess, meningitis, and lateral sinus thrombosis [3, 6, 10]. Subdural empyema, the purulent collection between the dura and arachnoid matter, is a rare but serious intracranial complications of COM [8, 11]. It is exceedingly rare that a study by Dongol et al. found only one case of subdural empyema (3.7%) out of 27 patients of chronic otitis media with intracranial complications [7]. Extension from otogenic infections is the most common cause of subdural empyema in older children [8, 9, 12]. The most common pathogens are *Streptococcus pneumoniae* (most common), *Haemophilus influenzae*, *Moraxella catarrhalis*, *Staphylococcus aureus*, Gram-negative rods (*Pseudomonas aeruginosa*, *Escherichia coli*, *Proteus*, *Klebsiella*) as well as anaerobic bacteria such as Bacteroides, Fusobacterium, Prevotella and Actinomyces species [5, 13–15]. Patients with Subdural empyema present with fever, headache, vomiting, and altered mental status [11, 14, 16, 17]. MRI and CT scan are important in the diagnosis of subdural empyema; MRI being the most sensitive one [6, 8, 12]. Culture and sensitivity from the pus is necessary although the yield is low [5–8, 14]. The principles of treatment, common to all intracranial complications, include early definitive diagnosis, appropriate systemic antibiotic therapy, neurosurgical intervention, and treatment of the ear lesion after the patient stabilizes.

## Case presentation

An 8 years old female patient presented with the complaint of right ear purulent discharge and right post auricular swelling of 2 weeks’ period and intermittent high-grade fever and altered mental status of 3 days’ duration prior to her admission. She had history of recurrent purulent discharge from her right ear for almost 2 years. The most recent of these occurrences was 2 weeks before her admission; at which time her parents had brought her to the pediatric hospital from where she was subsequently referred to the ENT department. In the ENT department, she was diagnosed with chronic otitis media and was prescribed amoxicillin (250 mg PO q8hr) for 7 days and was appointed for surgical intervention after 3 months. However, she was brought back to the pediatric ER after 2 weeks as her condition had deteriorated. The patient is born to a low-class nomadic family from the Rashaida ethnic group that lives in a small village near a coastal town of Eritrea. The family is an extended family of 15 people whose economy is dependent on pastoralism. Apart from the recurrent otitis media, the patient’s past medical history reveals no pertinent dental or other medical illnesses. She also had no history of trauma. According to the parents, the child was fully vaccinated. Moreover, the family have no history of relevant past medical illnesses.

**Fig. 1:**
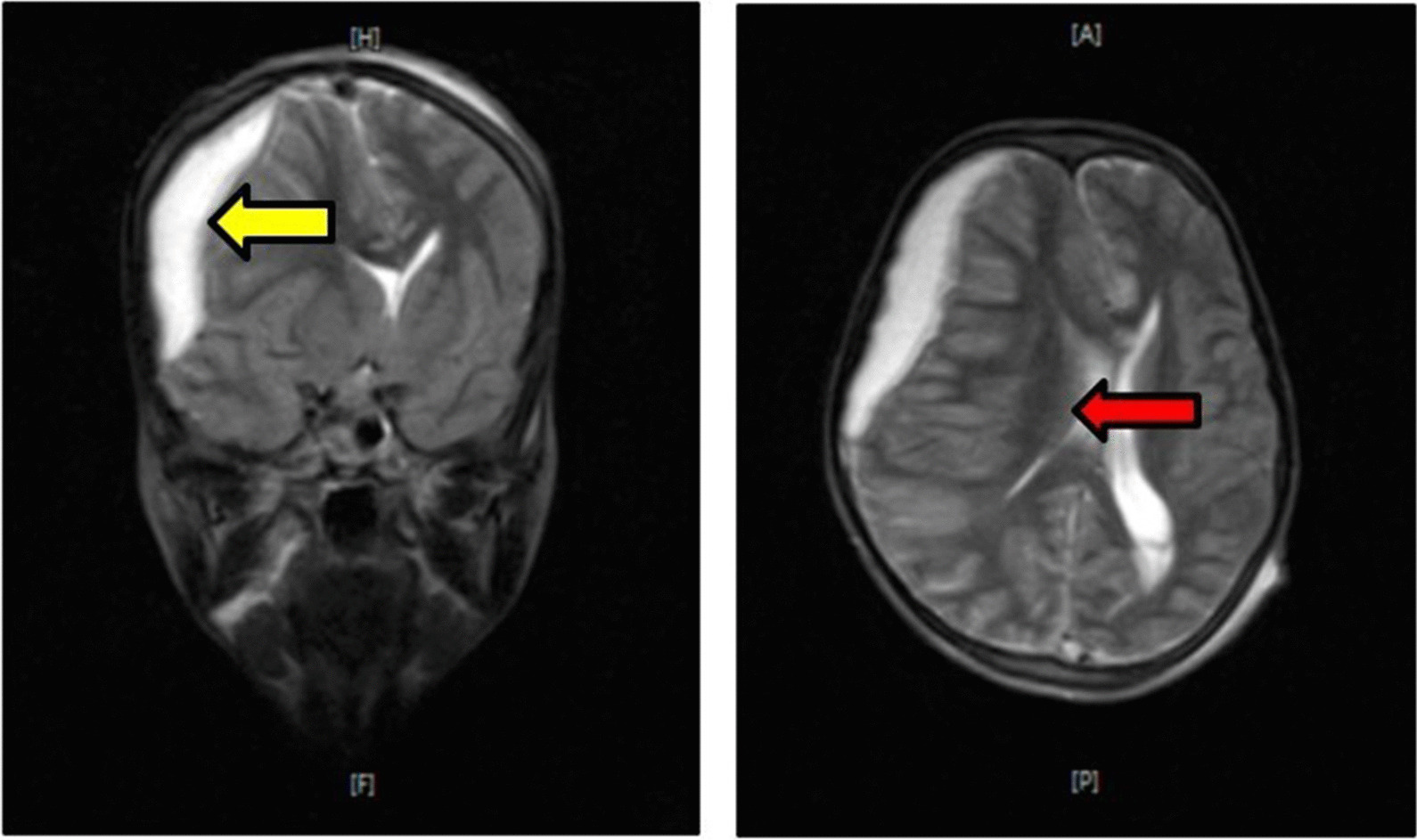
MRI images of the patient. Figure clearly shows a right fronto-temporal-parietal subdural empyema (yellow arrow) effacing the adjacent cortical sulci and compressing the right lateral ventricle, midline shift to the left (red arrow)

On physical examination, the patient was confused. She was tachycardic, tachypnic and febrile: otherwise, the other vital signs were within the normal range (BP-100/70, PR-114, RR-28, T°-38.5 °C, SPO_2_–94%). She had purulent foul-smelling discharge from the right ear. There was a 3 cm by 4 cm swelling in the postauricular area which was erythematous and had a 0.5 cm by 0.4 cm opening with a serosanguinous discharge over the most fluctuant part of the swelling. Examination of the chest, cardiovascular system, abdomen, and extremities showed no significant finding. Her neurological examination on admission, however, revealed that she had a Glasgow Coma Scale (GCS) of 13/15. The cranial nerves were grossly intact. Muscle strength was of normal power (5/5) throughout and the tone was with in normal limit. Deep tendon reflexes were symmetric and of normal grade (2+). Sensory examination showed that it was intact. Examination of the meningeal signs showed positive nuchal rigidity but negative Kernig’s and Brudziński’s signs. She was admitted to the Pediatric ICU with the diagnosis of sepsis, right ear chronic suppurative otitis media, acute mastoiditis, and right post-auricular subperiostial abscess. She was started on empiric antibiotics: Metronidazole (7.5 mg/Kg IV q8hrs) and Ceftriaxone (50 mg/Kg IV q12hrs). Five hours following her admission, her GCS lowered to 10/15 and 12 h later to 6/15. Thus, a diagnosis of brain abscess was entertained; however, MRI could not be done right away to confirm the diagnosis. On the 3rd day of her admission, her GCS was 3/15 and her right pupil was dilated and nonreactive to light while the left pupil was midway dilated with sluggish reaction to light. Full blood count was done and revealed Hgb of 6 g/dL, WBC count of 22.3 × 10^3^/micL with 80.8% Neutrophils. She was transfused with 2 units of blood (PRBC). Up on her admission to the Pediatric ICU, despite the patient’s altered level of consciousness, lumbar puncture (LP) was decided to be necessary. Even though, the patient had altered level of consciousness, she had none of the lateralizing signs (no signs of focal neurological deficit) nor did she have any of the Cushing’s triad (an increased systolic blood pressure, bradycardia, and an abnormal respiratory pattern) that indicate the possibility of an increased intracranial pressure. And during her arrival, MRI/CT scan could not be done nor were there any other ways the intracranial pressure could be measured. Moreover, as the patient had nuchal rigidity during admission along with the altered mental status, meningoencephalitis was one of the top differentials. These were the reasons LP was decided to be done in the presence of altered level of consciousness, before the patient was started on any antibiotics. CSF analysis was normal with no blood cells or organisms present under microscopic analysis, a glucose level of 65 mg/dL and a total protein level of 0.2 g/L. Culture of the pus taken from the right ear and the right postauricular area had no growth; thus, she continued her treatment with Metronidazole and Ceftriaxone. MRI was done on day 3 of her admission and showed right fronto-tempo-parietal subdural empyema with mass effect, effacing the adjacent cortical sulci and compressing the right lateral ventricle, shifting the midline to the left as presented in Fig. 1. Once the neurosurgical team were consulted, they decided to do an emergency craniotomy. In the OR an estimated amount of 30 ml of pus was removed from the subdural space. Incision and drainage of the post auricular subperiostial abscess was also done intraoperatively. Vancomycin (15 mg/Kg IV q6hrs) and Chloramphenicol ear drops were added to her antibiotic regimen for 14 days. Preoperatively she was also given mannitol to lower the intracranial pressure until the surgical intervention was done. The patient showed marked improvement post-operatively. She became fully conscious with GCS 15/15 and was communicating well by post-op day 4. After the neurosurgical aspects of the illness resolved, the patient was attached to the ENT department. However, no further information on the outcome could be attained as the patient was lost to follow-up after she was discharged from the pediatric hospital.

The CARE checklist was used to appropriately report the findings of this case study. (Additional file 1).

## Discussion

Subdural empyema is a rare and fatal intracranial complication of chronic otitis media with a mortality rate of 12.2% to 28% [11, 18]. The predisposing risk factors include neglected middle ear infections, lack of access to health care, immunosuppression, and poor economic status. Moreover, the lack of access to imaging modalities such as CT scan and/or MRI delay the diagnosis of subdural empyema and therefore increase the mortality rate [7, 10, 19, 20]. Over the past years, the incidence of complications of otitis media have decreased with the use of antibiotics, improved imaging studies, the introduction of the pneumococcal vaccine, and multidisciplinary management approach [6, 7].

### Clinical presentation

The symptoms and signs in patients with subdural empyema are due to both the pus collection and the mass effect on the brain. The classical triad of presentation which include headache, fever and vomiting is present only in 36% to 50% of the patients. This shows that the triad is not very sensitive in diagnosing subdural abscess. To emphasise this fact, as was already mentioned above in the case study, the patient had only fever from the so called ‘classic triad’. Therefore, it is generally agreed that the symptom complex of fever, headache, vomiting, and altered mental status is rather more precise [8, 17, 21]. Even with this, due to the rarity of the condition, the diagnosis is often delayed if not missed. Therefore, physicians need to have a high index of suspicion in patients who have history of COM.

### Investigation

Radio-imaging using CT scan or MRI is required to confirm the diagnosis. In the early stages of the disease however, CT scan can be normal in 63% of the cases [8]. As MRI can identify intracranial edema better than CT scan, it is more preferable to use MRI for earlier diagnosis of intracranial complication [6]. Similarly MRI is better in recognizing the extension of the empyema into the interhemispheric fissure and the posterior fossa [14]. Hence, for accurate diagnosis of suspected cases of intracranial subdural empyema, MRI is the gold standard with a sensitivity of 93% [8, 12]. Laboratory analysis of blood shows elevated WBC, ESR, and C-reactive protein [6]. Culture and sensitivity of intraoperative pus should be done, even though the empyema is sterile and has no yield in 50% of the cases [6, 8, 17]. However, it is important to note that anaerobic bacteria are common pathogens of subdural empyema. In our case, the laboratory in the hospital lacked culture media for anaerobic bacteria, which could also be a possible reason to as why the CSF, nor the pus from the craniotomy showed no growth. Therefore, we suggest that culture for anaerobic bacteria not to be overlooked. Another reason for culture with no yield could also be the administration of antibiotics before any sample is taken.

### Management

The mainstay treatment of subdural empyema includes the use of antibiotics and surgical evacuation of the pus. There should be immediate initiation of broad-spectrum antibiotics. A combination of oxacillin/nafcillin, a third-generation cephalosporin and metronidazole cover the most common pathogens. Due to the increasing presence of penicillin resistant *Streptococcus pneumoniae* and *Staphylococcus aureus* the use of vancomycin instead of oxacillin is preferred [6, 8, 16, 17, 22]. This was done in our case and the outcome was satisfactory. Most recommend a minimum 2 weeks of IV antibiotics while others suggest continuation for 6 weeks. In our case, the 2 weeks of IV antibiotics were found to be adequate. But almost all agree that IV antibiotics must be followed by 6 weeks of oral antibiotics [6, 8, 16, 23].

Surgical drainage of the subdural pus is the next crucial step following the initiation of antibiotics. The surgical procedure can either be craniotomy or burr whole. Craniotomy is preferred as it allows adequate exploration; effective decompression of increased intracranial pressure and complete removal of the pus, loculations as well as the possible calcifications on the abscess wall [8, 11, 14, 17]. Moreover, recurrence of subdural empyema is more common in Burr hole (almost 40%) as compared to craniotomy [16, 17, 21]. However, in the weak patients such as those in septic shock or when the empyema is parafalcine, Burr hole is the preferred procedure. The surgical intervention should be done as soon as possible, since any delay results in worsening of outcome. Disability reaches as high as 70% in those who were operated after 72 h of presentation as compared to the 10% in those who were operated within 72 h [11, 16, 18]

Administration of prophylactic anticonvulsants is advised as seizures occur in 32% to 44% of patients with subdural empyema [16, 17, 21]. Moreover mannitol should be given preoperatively and postoperatively to reduce the increased intracranial pressure [6, 16, 22]. After the patient stabilizes, the chronic otitis media and mastoiditis should be properly treated by the ENT specialists [3, 6, 10]. Similarly, our patient was later on transferred to the ENT department where her chronic otitis media was properly addressed.

Factors such as age of the patient, delay in presentation, extent of the sepsis, and the subdural empyema along with level of consciousness at presentation significantly affect the outcome of the patient [17]. Morbidities such as sensorineural hearing loss, residual neurological deficits including seizures and hemiparesis are seen in about 28% of the survivors [3, 11]. Therefore to achieve good results early diagnosis, immediate surgical intervention, and appropriate antibiotic therapy are of paramount importance.

## Conclusion

Patients with a positive history of chronic otitis media or any otologic complaints such as otorrhea who present with fever, headache, vomiting and altered level of consciousness should trigger a high index of suspicion for subdural empyema. MRI is the gold standard to confirm the diagnosis. Early diagnosis and timely surgical intervention accompanied by appropriate antibiotic therapy are of utmost significance in the management of subdural empyema following chronic otitis media.

### Supplementary Information

Additional file 1. CARE reporting checklist.

## Data Availability

The complete dataset used and/or analyzed during the current study are available from the corresponding authors and can be accessed upon a reasonable request.
